# Association between Adipose Tissue Depots and Dyslipidemia: The KORA-MRI Population-Based Study

**DOI:** 10.3390/nu14040797

**Published:** 2022-02-14

**Authors:** Ricarda von Krüchten, Roberto Lorbeer, Katharina Müller-Peltzer, Susanne Rospleszcz, Corinna Storz, Esther Askani, Charlotte Kulka, Christopher Schuppert, Wolfgang Rathmann, Annette Peters, Fabian Bamberg, Christopher L. Schlett, Blerim Mujaj

**Affiliations:** 1 Department of Diagnostic and Interventional Radiology, Medical Center, Faculty of Medicine, University of Freiburg, 79106 Freiburg, Germany; ricarda.kruechten@uniklinik-freiburg.de (R.v.K.); katharina.mueller-peltzer@uniklinik-freiburg.de (K.M.-P.); esther.askani@uniklinik-freiburg.de (E.A.); charlotte.kulka@uniklinik-freiburg.de (C.K.); fabian.bamberg@uniklinik-freiburg.de (F.B.); christopher.schlett@uniklinik-freiburg.de (C.L.S.); 2 Department of Radiology, Ludwig-Maximilians-University Hospital, 80336 Munich, Germany; roberto.lorbeer@med.uni-muenchen.de; 3 Chair of Epidemiology, Institute for Medical Information Processing, Biometry and Epidemiology, Medical Faculty, Ludwig-Maximilians-University, 81377 Munich, Germany; susanne.rospleszcz@helmholtz-muenchen.de (S.R.); peters@helmholtz-muenchen.de (A.P.); 4 Institute of Epidemiology, Helmholtz Zentrum München, German Research Center for Environmental Health, 85764 Neuherberg, Germany; 5 Department of Neuroradiology, Medical Center, Faculty of Medicine, University of Freiburg, 79106 Freiburg, Germany; corinna.storz@uniklinik-freiburg.de; 6 Department of Diagnostic and Interventional Radiology, Heidelberg University Hospital, 69120 Heidelberg, Germany; christopher.schuppert@med.uni-heidelberg.de; 7 German Diabetes Center, Institute for Biometrics and Epidemiology, 40225 Düsseldorf, Germany; rathmann@ddz.de; 8 German Center for Diabetes Research (DZD), Partner Site Neuherberg, 85764 Neuherberg, Germany

**Keywords:** adipose tissue, total cholesterol, LDL-cholesterol, HDL-cholesterol, triglycerides, magnetic resonance imaging, population-based cohort

## Abstract

Obesity increases the risk of cardiovascular diseases (CVD), however, whether adipose tissue relates to dyslipidemia, and consequently to cardiovascular events remains unknown. Thus, we investigated the association of adipose tissue with circulating lipoproteins and triglycerides (TG) in subjects without CVD. 384 participants from the KORA-MRI study (mean age 56.2 ± 9.2 years; 41.9% female) underwent whole-body 3T-MRI. Visceral (VAT) and subcutaneous adipose tissue (SAT) derived from T1-DIXON-sequence using a semi-automatic algorithm. Total cholesterol, high-density lipoprotein (HDL), low-density lipoprotein (LDL), and TG were measured. Linear regression was applied to examine the relationships between adipose tissue, circulating lipoproteins, and TG, adjusting for risk factors. VAT was associated with total cholesterol (per SD increase) (ß = 0.39, *p* < 0.001). Total adipose tissue (TAT) and VAT were inversely associated with HDL (ß = −0.09, *p* = 0.009; ß = −0.14, *p* < 0.001), and positively associated with LDL (ß = 0.32, *p* < 0.001; ß = 0.37, *p* < 0.001). All adipose tissues were associated with TG (ß = 0.20, *p* < 0.001; ß = 0.27, *p* < 0.001; ß = 0.11, *p* = 0.004). Stratified analysis by sex and body mass index (BMI) was confirmatory in women and in individuals with BMI < 30. Our results suggest that adipose tissue plays an important role in increasing CVD risk independent of BMI, whereas gender imbalance may be explained by accurate characterization and quantification of adipose tissue.

## 1. Introduction

Obesity is a major risk factor for cardiovascular diseases (CVD), leading to premature death [1,2]. Obesity, characterized by the deposition of adipose tissue stored as visceral adipose tissue (VAT) and subcutaneous adipose tissue (SAT) [3], has been linked to metabolic disorders, hypertension, and type-2 diabetes mellitus, which additionally contribute to an increased risk of cardiovascular disease. [4]. On the other hand, dyslipidemia with profiles of elevated levels of low-density lipoprotein (LDL) [5,6], total cholesterol, triglycerides, and low levels of high-density lipoprotein (HDL) [7], are the leading causes of CVD, especially of atherosclerotic etiology [5,8,9].

The accumulated adipose tissue depots at various body locations further exhibit secretory functions and exert inflammatory effects [10]. VAT rather than SAT comprise a broad spectrum of pro-inflammatory mediators, a higher endocrine function, and insulin resistance [11,12]. Moreover, VAT has a greater lipolysis capacity generating free fatty acids (FFA), and VAT is predominantly associated with metabolic disorders and risk factors in obese individuals [13,14,15]. However, when considering body mass index (BMI) categories using anthropometric measurements to assess the adipose tissue, adipose tissue may not directly associate with CVD, known as the obesity paradox observed in obese patients compared to non-obese patients. A recent study reported that regional body fat distribution is associated with higher cardiovascular disease risk among postmenopausal women with normal BMI [16]. Similarly, the UK Biobank, a large cohort study, reported that different adipose measures were associated with an incidence of cardiovascular diseases independent of BMI [17].

Nowadays, various imaging techniques are in use for the assessment of adipose tissue, such as magnetic resonance imaging (MRI), dual-energy X-ray absorptiometry (DEXA), abdominal ultrasound, computed tomography (CT), or more simply anthropometric measurements [18]. The accumulated body fat may deteriorate existing risk factors of cardiovascular diseases, and the characterization and quantification of adipose tissue may also provide more knowledge on the possible indirect relation of adipose tissue on atherosclerotic CVD through interplay with dyslipidemia. However, evidence gaps remain and whether adipose tissue directly contributes to circulating dyslipidemia with worse lipid profile for cardiovascular diseases, where LDL cholesterol plays a central role, remains scarce. Nevertheless, MRI may provide more accurate adipose tissue characterization and quantification without exposure to ionizing radiation [3]. The study investigated the relationship between different adipose tissues and circulating lipoproteins and triglyceride levels in the population-based KORA-MRI study.

## 2. Materials and Methods

### 2.1. Study Population

The KORA-MRI study is a population-based cohort in the region of Augsburg, Germany [19]. Subjects aged 25–74 years, residents in the Augsburg region, were recruited and examined at the KORA study center between June 2013 and September 2014. A 3-Tesla whole-body MRI scan was incorporated in the KORA-MRI study [19]. Subjects were selected for an MRI scan if they met the following inclusion criteria, such as qualification of being in the prediabetes, diabetes, or control group, and were informed of study consent. The following exclusion criteria were applied: age > 74 years; participants with a known history of coronary artery disease, myocardial infarction, peripheral artery disease, stroke, and/or unavailable oral glucose test, pregnancy, poor overall health condition, or other physical limitations. Subjects with contraindications to MRI scan, such as known gadolinium contrast allergy, cardiac stents, cardiac pacemaker or implantable defibrillator, implanted metal parts, breast-feeding women, subjects with claustrophobia, and subjects with impaired renal function were excluded. From a total of MRI scans (*n* = 400) [20], subjects with incomplete measurements of VAT (*n* = 10) and SAT (*n* = 16) were excluded. Hence, 384 subjects were included in the analysis. All participants gave written informed consent. The KORA-MRI study was approved by the Institutional Research Ethics Board of the Medical Faculty of Ludwig-Maximilian University Munich and compiled with Helsinki’s declaration on human research [21].

### 2.2. Whole-Body MR Imaging

A 3-Tesla MRI scan (MagnetomSkyra, Siemens AG, Healthcare Sector, Erlangen, Germany) used an 18-channel body surface coil and a table-mounted spine matrix coil to perform the whole-body imaging. The protocol included sequences with the entire body (from neck to below hip) for tissue/organ quantification and particular organs, e.g., brain, carotid arteries, or fat compartments [19]. A 2-point DIXON T1 sequence, at submaximal inspiration breath-hold and an acquisition time of 15 s, was used to analyze the body adipose tissue volume. Slice thickness was 3 mm, coronal acquired, including a FOV of 488 × 716, a matrix of 256 × 256, a time of repetition (TR) of 4.06 ms, and a time of echo (TE) of 1.26 ms [19]. All images were analyzed by blinded independent experienced readers, unaware of the clinical characteristics of study subjects [19].

### 2.3. MR-Image Analysis for Adipose Tissue Depots

In a fat-selective tomogram (slice thickness 5 mm in 5-mm increments) based on the volume-interpolated, adipose tissue was calculated in three-dimensional fat images from the 2-point-DIXON sequence. An in-house algorithm based on Matlab R2013a was used to semi-automatically quantify total adipose tissue from the femoral head to the cardiac apex and if necessary, segmentations were manually adjusted [19]. VAT quantification was performed from the femoral head to the diaphragm and SAT quantification from the femoral head to the cardiac apex in the same way by each reader. Total adipose tissue (TAT) was calculated as the sum of VAT and SAT.

### 2.4. Assessment of Lipid Profile

Venous blood was collected from all study participants in fasting state, at examination visit and was shipped within 2 to 4 h to the laboratory of Augsburg Central Hospital. Total cholesterol and LDL cholesterol were measured using the Boehringer CHOD-PAP (Roche Diagnostics GmbH, Mannheim, Germany) assay, and HDL-cholesterol using the phosphotungstic acid method (Boehringer Mannheim, Mannheim, Germany). Triglycerides were measured with the Boehringer GPO-PAP assay (non-fasting in diabetic subjects) [19].

### 2.5. Other Risk Factors

Information on risk factors was recorded through physical examination, interview, and blood sampling after overnight fasting from all subjects at the study center. BMI and body surface area (BSA) were calculated based on height and weight, while smoking status, alcohol use (g/day), glucose-lowering, antihypertensive, lipid-lowering medication was assessed by questionnaire. Diabetes mellitus was defined according to the WHO criteria as follows:

- Prediabetes

Impaired glucose tolerance (normal fasting glucose concentration and a 2-h serum oral glucose tolerance test (OGTT): glucose concentration between 140 and 200 mg/dL; and/or an impaired fasting glucose concentration, as defined by fasting glucose levels between 110 and 125 mg/dL, and a normal 2-h serum glucose concentration).

- Diabetes

Two-h serum glucose concentration as determined by OGTT that was > 200 mg/dL and/or a fasting glucose level that was > 125 mg/dL).

Glycated hemoglobin (HbA1c) analyzed in hemolyzed whole blood using the cation-exchange high-performance liquid chromatographic, photometric VARIANT-II-TURBO HbA1c Kit-2.0 assay, or on a VARIANT-II-TURBO Hemoglobin Testing System (Bio-Rad Laboratories Inc., Hercules, CA, USA). Serum fasting glucose (FG) was sampled, and 75 g of anhydrous glucose (Dextro OGT; Boehringer Mannheim) was given to participants without a known diagnosis of type 2 diabetes or taking glucose-lowering agents. Serum FG was measured using an enzymatic colorimetric method (Dimension Vista 1500, Siemens Healthcare Diagnostics or Cobas c702, Roche Diagnostics). Serum fasting insulin (FI) levels were determined from blood samples using serum FI and were measured by a solid-phase enzyme-labeled chemiluminescent immunometric assay (Immulite 2000 Xpi, Siemens Healthcare Diagnostics) or by an electrochemiluminescence immunoassay (Cobas e 602, Roche Diagnostics GmbH). Hypertension was defined as systolic blood pressure > 140 mmHg, diastolic blood pressure > 90 mmHg, or receiving current antihypertensive treatment. Serum creatinine concentrations were analyzed using an enzymatic colorimetric method (Dimension Vista 1500, Siemens Healthcare Diagnostics or Cobas c702, Roche Diagnostics GmbH). Serum γ-glutamyltransferase (GGT), alkaline phosphatase (ALP), and aspartate aminotransferase (AST) levels were determined using a Merck Diagnostica kit (Merck, Whitehouse Station, NJ, USA) on an Elan Autoanalyzer (Merck) [19].

### 2.6. Statistical Analysis

The distribution of the study population characteristics for continuous and categorical variables or percentages was described by using mean and standard deviation (SD), median (interquartile ranges (IQRs)), or frequency with percentage, respectively. No missing data were on exposure, outcome, or covariates. Differences between women and men were tested by t-test, Wilcoxon rank-sum test, or chi2-test. A natural logarithmic transformation was performed to normalize the distribution of TAT, VAT, SAT, and triglycerides. A two-step approach investigated the association between TAT, VAT, and SAT with total cholesterol, HDL, LDL, and triglycerides. First, using linear regression analysis we investigated the association between TAT, VAT, SAT, and VAT/SAT ratio with total cholesterol, HDL, LDL, triglycerides (TG), and TG/HDL ratio. Model 1 was adjusted for sex and age. Model 2 was additionally adjusted for BSA, smoking, alcohol use, diabetes, and hypertension. Further, model 3 adjusted additionally for glucose, insulin, GGT, AST, ALP, and creatinine, while model 4 was additionally adjusted for lipid-lowering medication and physical activity. Second, in stratified analysis, we investigated differences for gender and BMI categories (<25, ≥25–30, and >30), while adjusting for the models mentioned above. All analyses were performed using Stata (Stata 16.1 Corporation, College Station, TX, USA).

## 3. Results

Table 1 summarizes the characteristics of the 384 subjects included in the analysis. The mean age was 56.2 ± 9.2 years and 41.9% of subjects were women. Among the subjects, 20.3% were current smokers, and mean alcohol use was 18.6 ± 24 g/day. Anthropometric measures such as BMI was 27.9 ± 4.7 kg/m^2^, waist circumference was 98 ± 13.8 cm, hip circumference was 106.6 ± 8.2 cm, and waist-to-hip ratio was 0.92 ± 0.09. In all subjects, the median TAT, VAT, and SAT were 11.9 L (8.57; 16.11), 4.03 L (2.43; 6.1), 7.12 L (5.44; 9.94), respectively. Details of the lipid profile of the study population are given in Table 2.

### 3.1. Association between Adipose Tissue with Lipid Profile

**Figure 1:**
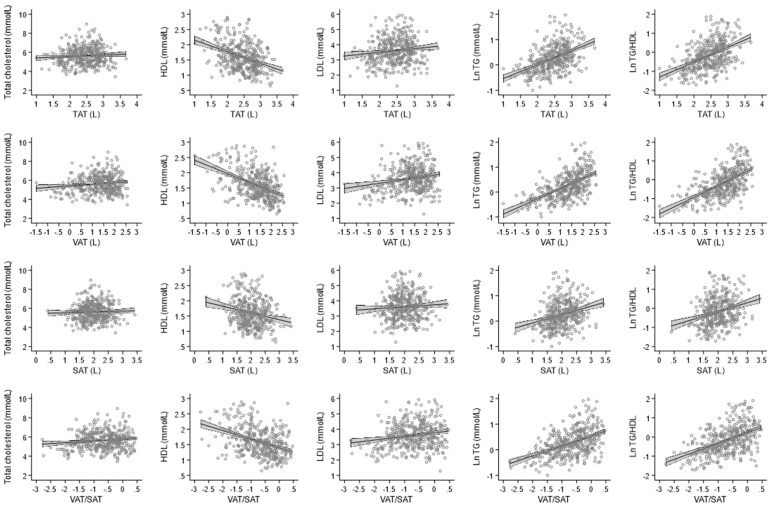
The relationship between natural log of TAT, VAT, SAT, and VAT/SAT ratio with total cholesterol, HDL, LDL, triglycerides, and triglycerides/HDL ratio. The scatter plots depicting the univariate regression line (continues line) and 95% confidence interval (outer lines with gray area).

We found a positive association between VAT and total cholesterol (per SD increase: ß = 0.15, *p* = 0.012) in a model adjusted for sex and age (Model 1 in Table 3 and Figure 1). The association remained significant after further adjustment for other risk factors in models 2, 3, and 4 (β = 0.39, *p* = <0.001 in model 4) (Table 3). In contrast, we found an inverse association between TAT, and VAT with HDL (TAT β = −0.09, *p* = 0.009, VAT β = −0.14, *p* = <0.001, respectively) (Table 3). While assessing LDL, we found an association between TAT, and VAT, similarly to HDL, but in opposite direction to HDL (TAT β = 0.32, *p* = <0.001, VAT β = 0.37, *p* = <0.001, respectively) (Table 3). Finally, TAT, VAT, and SAT were associated with triglycerides in model 1–3 and remained significant in the fully adjusted model 4 (TAT β = 0.20, *p* = <0.001, VAT β = 0.27, *p* < 0.001, SAT β = 0.11, *p* = 0.004, respectively) (Table 3). We did not find an association between TAT and SAT with total cholesterol and SAT and LDL. When the VAT/SAT ratio was assessed, the estimates were similar to VAT, proving that the association was driven by VAT. Also, all adipose tissue depots were significantly associated with TG/HDL ratio (Table 3).

### 3.2. Association between Adipose Tissue with Lipid Profile According to Sex

**Figure 2:**
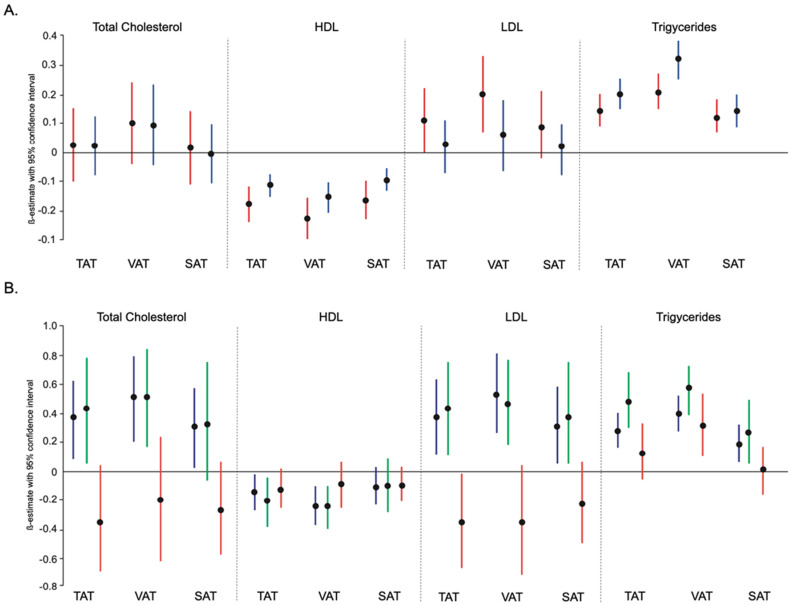
The relationship between TAT, VAT, SAT and total cholesterol, HDL, LDL, and triglycerides, according to gender (panel (**A**), red—females, blue—males) and BMI categories (<25 blue, ≥25–30 green, and > 30 red) (panel (**B**)). The univariate ß-estimate with 95% confidence interval depicts the effect size for each parameter.

When considering gender differences, in females we found an inverse association between TAT, and VAT with HDL (per SD increase: TAT β = −0.15, *p* = 0.012, VAT β = −0.19, *p* = 0.001 in model 4, respectively; Figure 2 and Table S2). Interestingly, only VAT was significantly associated with LDL and remained significant in all models (VAT β = 0.49, *p* = <0.001). Whereas for triglycerides, all adipose tissue parameters TAT, VAT, and SAT were significantly associated (TAT β = 0.20 *p* = <0.001, VAT β = 0.25, *p* = <0.001, SAT β = 0.14, *p* = 0.011, respectively) (Figure 2 and Table S1). When concentrating on males, we found an inverse association between VAT and HDL (VAT β = −0.10, *p* = 0.046; Table S3), but surprisingly we did not find an association between any adipose tissue depots with LDL. Similarly, we found an association between TAT and VAT with triglycerides in all models in females but did not find an association between SAT and triglycerides.

### 3.3. Association between Adipose Tissue with Lipid Profile according to Body Mass Index

In categories of BMI, in the low tertile category (BMI < 25) we found an association between VAT and total cholesterol (per SD increase: VAT β = 0.32, *p* = 0.032 in model 4). Also, we found an inverse association between TAT, and VAT with HDL (TAT β = −0.18, *p* = 0.005, VAT β = −0.25, *p* < 0.001), an association between VAT and LDL (β = 0.42, *p* = 0.003), and for triglycerides associations with all three adipose tissue parameters TAT, VAT, and SAT (Figure 2 and Table S4). Further, in the middle tertile category (BMI ≥ 25–30) we were able to confirm findings similar to low tertile, whereas, in the high tertile category (BMI > 30), the results were not significant (Figure 2 and Tables S5 and S6).

## 4. Discussion

This population cohort study found that VAT was positively associated with total cholesterol, LDL, and triglycerides, and inversely associated with HDL, while the SAT was associated with triglycerides only. Interestingly, the findings replicated in females, but not in males, and categories with normal weight, and overweight, but not in obese subjects.

Traditionally, the VAT has been considered the more pathogenic adipose tissue than SAT, but no study has investigated the role of total adipose tissue volume related to circulating lipoprotein. Previous studies have implicated that the VAT and SAT were associated with cholesterol lipoproteins and triglycerides [22] and overall metabolic risk in obese and non-obese subjects [12,13,14]. The Framingham Heart Study VAT and SAT were associated with the overall adverse metabolic risk profile, including high systolic and diastolic blood pressure, impaired glucose levels, or metabolic syndrome [13]. In addition, the study concluded that both VAT and SAT contributed equally to dyslipidemia with high levels of total cholesterol, triglycerides, and low HDL levels. Our results are in line with the Framingham Heart Study, which indicated that VAT contributed to higher circulatory lipoproteins in relation to total cholesterol and HDL, but conflicting when SAT is in consideration. In our study, SAT was not associated with the mentioned parameters but only with triglycerides. Although differences exist between study populations in VAT and SAT measurements, the BMI was revealed to be similar.

Similarly, a Japanese study with healthy employees assessing VAT, and SAT reported that VAT affected HDL cholesterol and triglycerides, but not SAT [22]. Another study with Japanese Americans reported that VAT change independently influenced atherogenic dyslipidemia, not SAT [23]. Furthermore, the Hitachi Health Study reported that VAT change was associated with atherogenic lipid levels [24]. Our results are consistent with studies that consider VAT atherogenic and affect lipoprotein and triglyceride levels, but not SAT. The results support the findings of SAT to be associated with triglyceride levels only. In addition, elaboration of the impact of TAT, the TAT associated with LDL, and inversely with HDL, similar to VAT but not to SAT, suggesting that VAT was the main driver of the association within TAT not SAT. Therefore, TAT may not be a reliable parameter for adipose tissue characterization assessment.

The metabolic effects of VAT mediated via the release of free fatty acids into the systemic circulation, leading to dyslipidemia [25,26]. Such effect of VAT could be explained by highly innervated β-adrenergic receptors that exhibit greater lipolytic activity compared to SAT [27] and thus mobilize free fatty acids into the portal circulation. In this context, with centralized production of cholesterol through the liver [28], we additionally adjusted for the use of lipid-lowering treatments [29] and observed no impact of lipid-lowering treatment in relation to adipose tissue to circulatory lipoproteins and triglycerides. Atherogenic profile of dyslipidemia with increased LDL and decreased HDL had been observed with higher amounts of VAT, and the VAT was an independent predictor of future atherogenic dyslipidemia [23]. Thus, we hypothesize that VAT contributes to higher cardiovascular risk through increased LDL, which later plays a central role in the initiation and progression of atherosclerosis [30]. Furthermore, the SAT seems to relate more to triglycerides, and our finding is in line with results from a Swedish study cohort that attributed SAT as an essential contributor to elevated triglycerides [31].

Additionally, elaborating the “obesity paradox,” the UK Biobank study reported a J-shaped association when considering categories of BMI in relation to a higher incidence of CVD [17]. The BMI confounding and obesity paradox observed in our results is also within the category of obese subjects. This highlights that BMI is an important confounder in relation to risk assessment for CVD of atherosclerotic origin, where adipose tissue may play an important role. Thus, future implementation of MRI adipose tissue characterization and quantification as a standard procedure of whole-body MRI examination may provide better estimates of adipose tissue and can be used in risk assessment for CVD, especially of atherosclerotic etiology.

Strengths of the study incorporate the implementation of advanced 3T whole-body MRI technology with detailed protocol, which included three-dimensional fat images from the 2-point-DIXON sequences. Adipose tissue assessment used semi-automatic quantification based on in-house algorithms, also. Our study recruited participants from a population-based cohort with healthy individuals free of cardiovascular diseases. Moreover, multilevel testing was applied to confirm our results through confirmation in gender and BMI categories. Nonetheless, the study encounters a few limitations necessary to mention. First, cross-sectional design study limits to draw causal inferences and generated hypotheses require further confirmation in other designs and populations. Second, our study represents a relatively small number of participants from the population-based cohort. Third, our study included only European descents, and the generalizability of findings may be limited to other populations or geographical regions.

## 5. Conclusions

Adipose tissue is associated with circulating lipoproteins, triglycerides, and consequently worse lipid profile. Irrespective of BMI, as an important confounder, adipose tissue may play an essential role in the increased risk of CVD and gender imbalance explained through characterization and quantification of the adipose tissue. Further studies are warranted, in a longitudinal design, to confirm our findings.

## Figures and Tables

**Table 1:** Study population characteristics.

	All	Female	Male	*p* Value
	*n* = 384	*n* = 161	*n* = 223	
Age, years	56.2 (±9.2)	56.3 (±8.9)	56.2 (±9.4)	0.929
Women	161 (41.9%)	-	-	
Body mass index, kg/m^2^	27.9 (±4.7)	27.4 (±5.3)	28.3 (±4.2)	0.081
Body surface area, m^2^	1.95 (±0.21)	1.78 (±0.16)	2.07 (±0.16)	<0.001
Waist circumference, cm	98 (±13.8)	91.3 (±13.7)	102.9 (±11.7)	<0.001
Hip circumference, cm	106.6 (±8.6)	106.5 (±10.2)	106.6 (±7.2)	0.921
Waist-to-hip ratio	0.92 (±0.09)	0.86 (±0.08)	0.96 (±0.07)	<0.001
Smoking status				0.083
Never	141 (36.7%)	68 (42.2%)	73 (32.7%)	
Past	165 (43%)	59 (36.7%)	106 (47.5%)	
Current	78 (20.3%)	34 (21.1%)	44 (19.7%)	
Alcohol use, (g/day)	18.6 (±24)	8.5 (±13.8)	25.9 (±27)	<0.001
Physical activity	229 (59.6%)	103 (64%)	126 (56.5%)	0.141
Diabetes	52 (13.5%)	13 (8.1%)	39 (17.5%)	0.008
HbA1c, %	5.57 (±0.72)	5.54 (±0.51)	5.59 (±0.84)	0.463
Fasting glucose, mmol/L	5.50 (5.11;6.11)	5.27 (4.88;5.88)	5.66 (5.27;6.27)	<0.001
Fasting insulin, pmol/L	54.6 (37.2;81.0)	49.9 (34.8;74.9)	60.0 (38.4;87.1)	0.004
Antidiabetic medication	31 (8.1%)	11 (6.8%)	20 (9%)	0.448
Hypertension	128 (33.3%)	45 (28%)	83 (37.2%)	0.057
Systolic, mm/Hg	120.6 (±16.4)	113.2 (±14)	126 (±15.9)	<0.001
Diastolic, mm/Hg	75.3 (±9.9)	72.1 (±8.5)	77.7 (±10.2)	<0.001
Antihypertensive medication	94 (24.5%)	43 (26.7%)	51 (22.9%)	0.388
Total cholesterol, mmol/L	5.63 (±0.94)	5.67 (±0.88)	5.60 (±0.97)	0.463
HDL, mmol/L	1.60 (±0.46)	1.82 (±0.46)	1.44 (±0.39)	<0.001
LDL, mmol/L	3.60 (±0.84)	3.54 (±0.81)	3.65 (±0.86)	0.199
Triglycerides, mmol/L	1.22 (0.88;1.78)	1.07 (0.77;1.37)	1.42 (0.99;2.13)	<0.001
Ratio TG/HDL	0.78 (0.48;1.37)	0.58 (0.42;0.91)	1.02 (0.61;1.75)	<0.001
Lipid lowering medication	41 (10.7%)	17 (10.6%)	24 (10.8%)	0.949
GGT, U/L	28.8 (17.4;45)	19.8 (13.8;31.8)	35.4 (24;57)	<0.001
AST, U/L	22.8 (19.2;28.8)	19.8 (16.8;25.8)	25.2 (21;30)	<0.001
ALP, U/L	27 (19.8;37.2)	21 (16.2;28.2)	31.2 (25.2;40.8)	<0.001
Serum creatinine, µmol/L	78.1 (±13.7)	68.3 (±10.5)	85.1 (±11.2)	<0.001
TAT, L	11.9 (8.57;16.11)	10.79 (7.3;16.17)	12.03 (9.18;16.11)	0.039
VAT, L	4.03 (2.43;6.1)	2.69 (1.39;4.06)	5.42 (3.47;7.32)	<0.001
SAT, L	7.12 (5.44;9.94)	8.41(5.74;11.79)	6.66 (5.37;8.72)	<0.001
Ratio VAT/SAT	0.52 (0.31;0.81)	0.29 (0.21;0.39)	0.74 (0.58;0.96)	<0.001

The values represent mean ± standard deviation (SD), median (interquartile ranges) or frequency along with percentage (%). *p* = *p*-value for difference based on t-test, wilcoxon rank-sum test or chi2-test, respectively; Abbreviations: AST = aspartate aminotransferase; ALP = alkaline phosphatase; GGT = γ-glutamyl transferase; HbA1c = Glycated hemoglobin A1c; HDL = high-density lipoprotein; LDL = low-density lipoprotein; SAT = subcutaneous adipose tissue; TAT = total adipose tissue; VAT = visceral adipose tissue.

**Table 2:** Lipid profile of study population according to tertiles of TAT, VAT, SAT and the ratio of VAT/SAT.

TAT	Low Tertile	Medium Tertile	High Tertile	*p* Value
Total cholesterol	5.49 (±0.89)	5.74 (±0.98)	5.64 (±0.92)	0.261
HDL	1.78 (±0.49)	1.60 (±0.45)	1.42 (±0.36)	<0.001
LDL	3.44 (±0.83)	3.71 (±0.87)	3.66 (±0.81)	0.031
Triglycerides	0.89 (0.73; 1.20)	1.27 (0.99;1.80)	1.58 (1.19; 2.30)	<0.001
Ratio TG/HDL	0.52 (0.38; 0.83)	0.86 (0.53; 1.35)	1.19 (0.7; 1.94)	<0.001
**VAT**				
Total cholesterol	5.46 (±0.94)	5.78 (±0.84)	5.64 (±1.00)	0.202
HDL	1.84 (±0.48)	1.58 (±0.37)	1.38 (±0.39)	<0.001
LDL	3.40 (±0.84)	3.76 (±0.76)	3.65 (±0.88)	0.015
Triglycerides	0.88 (0.73; 1.20)	1.26 (0.97; 1.60)	1.75 (1.25; 2.50)	<0.001
Ratio TG/HDL	0.50 (0.38; 0.71)	0.82 (0.54; 1.17)	1.40 (0.76; 2.28)	<0.001
**SAT**				
Total cholesterol	5.47 (±0.9)	5.81 (±0.96)	5.61 (±0.92)	0.292
HDL	1.71 (±0.48)	1.57 (±0.44)	1.52 (±0.43)	0.002
LDL	3.43 (±0.85)	3.79 (±0.82)	3.59 (±0.82)	0.179
Triglycerides	0.99 (0.77; 1.40)	1.27 (0.97; 2.00)	1.45 (1.09; 2.00)	<0.001
Ratio TG/HDL	0.61 (0.41; 1.01)	0.83 (0.53; 1.42)	1.00 (0.61; 1.56)	<0.001
**VAT/SAT ratio**				
Total cholesterol	5.54 (±0.84)	5.68 (±0.96)	5.66 (±0.99)	0.444
HDL	1.85 (±0.47)	1.53 (±0.40)	1.42 (±0.38)	<0.001
LDL	3.44 (±0.78)	3.71 (±0.84)	3.66 (±0.89)	0.055
Triglycerides	0.96 (0.75; 1.27)	1.28 (0.98; 1.75)	1.56 (1.06; 2.32)	<0.001
Ratio TG/HDL	0.52 (0.36; 0.78)	0.85 (0.56; 1.32)	1.19 (0.70; 1.96)	<0.001

Values are represented as mean with standard deviation (SD) or median with interquartile range (IQR); P- depicts for trend. Abbreviation: HDL = high-density lipoprotein; LDL = low-density lipoprotein; SAT = subcutaneous adipose tissue; TAT = total adipose tissue; TG = triglycerides; VAT = visceral adipose tissue.

**Table 3:** Association between TAT, VAT, SAT and VAT/SAT ratio with total cholesterol, HDL, LDL, triglycerides, and TG/HDL ratio.

Per SD Log Transformed	Model 1	*p*-Value	Model 2	*p*-Value	Model 3	*p*-Value	Model 4	*p*-Value
				**Total Cholesterol**				
TAT	0.05 (−0.04; 0.15)	0.294	0.35 (0.21; 0.49)	<0.001	0.32 (0.16; 0.49)	<0.001	0.35 (0.20; 0.51)	<0.001
VAT	0.15 (0.03; 0.27)	0.012	0.41 (0.26; 0.55)	<0.001	0.36 (0.20; 0.52)	<0.001	0.39 (0.23; 0.55)	<0.001
SAT	0.03 (−0.07; 0.12)	0.598	0.31 (0.17; 0.45)	<0.001	0.29 (0.13; 0.45)	<0.001	0.30 (0.15; 0.45)	<0.001
VAT/SAT ratio	0.07 (−0.06; 0.21)	0.294	0.50 (0.31; 0.70)	<0.001	0.46 (0.23; 0.69)	<0.001	0.51 (0.28; 0.73)	<0.001
	**HDL**							
TAT	−0.16 (−0.20; −0.12)	<0.001	−0.14 (−0.20; −0.09)	<0.001	−0.09 (−0.15; −0.02)	0.009	−0.09 (−0.16; −0.02)	0.009
VAT	−0.21 (−0.26; −0.16)	<0.001	−0.18 (−0.24; −0.12)	<0.001	−0.13 (−0.20; −0.07)	<0.001	−0.14 (−0.20; −0.07)	<0.001
SAT	−0.14 (−0.18; −0.10)	<0.001	−0.10 (−0.16; −0.04)	0.001	−0.04 (−0.10; 0.03)	0.273	−0.04 (−0.10; 0.03)	0.273
VAT/SAT ratio	−0.23 (−0.29; −0.17)	<0.001	−0.21 (−0.29; −0.13)	<0.001	−0.13 (−0.22; −0.03)	0.009	−0.13 (−0.22; −0.03)	0.009
	**LDL**							
TAT	0.09 (0.00; 0.17)	0.048	0.30 (0.17; 0.43)	<0.001	0.29 (0.14; 0.44)	<0.001	0.32 (0.18; 0.46)	<0.001
VAT	0.18 (0.07; 0.28)	0.001	0.36 (0.23; 0.49)	<0.001	0.34 (0.19; 0.49)	<0.001	0.37 (0.23; 0.52)	<0.001
SAT	0.07 (−0.02; 0.15)	0.114	0.26 (0.13; 0.39)	<0.001	0.25 (0.10; 0.39)	0.001	0.26 (0.12; 0.40)	<0.001
VAT/SAT ratio	0.12 (0.00; 0.25)	0.048	0.43 (0.25; 0.61)	<0.001	0.42 (0.21; 0.63)	<0.001	0.46 (0.25; 0.66)	<0.001
	**Triglycerides**							
TAT	0.23 (0.18; 0.28)	<0.001	0.31 (0.24; 0.37)	<0.001	0.20 (0.12; 0.27)	<0.001	0.20 (0.12; 0.27)	<0.001
VAT	0.33 (0.27; 0.38)	<0.001	0.36 (0.29; 0.43)	<0.001	0.26 (0.19; 0.34)	<0.001	0.27 (0.19; 0.34)	<0.001
SAT	0.18 (0.13; 0.23)	<0.001	0.23 (0.16; 0.30)	<0.001	0.11 (0.04; 0.19)	0.003	0.11 (0.04; 0.19)	0.004
VAT/SAT ratio	0.33 (0.26; 0.39)	<0.001	0.44 (0.34; 0.53)	<0.001	0.28 (0.17; 0.39)	<0.001	0.28 (0.17; 0.39)	<0.001
				**Ratio TG/HDL**				
TAT	0.33 (0.27; 0.39)	<0.001	0.39 (0.30; 0.48)	<0.001	0.24 (0.14; 0.34)	<0.001	0.24 (0.14; 0.35)	<0.001
VAT	0.46 (0.38; 0.54)	<0.001	0.47 (0.38; 0.56)	<0.001	0.33 (0.23; 0.44)	<0.001	0.34 (0.23; 0.44)	<0.001
SAT	0.27 (0.21; 0.34)	<0.001	0.28 (0.19; 0.38)	<0.001	0.13 (0.02; 0.23)	0.016	0.12 (0.02; 0.23)	0.018
VAT/SAT ratio	0.47 (0.38; 0.56)	<0.001	0.56 (0.43; 0.69)	<0.001	0.34 (0.20; 0.49)	<0.001	0.34 (0.19; 0.49)	<0.001

The beta estimate from linear regression model given with a 95% confidence interval represents the estimate size per SD of TAT, VAT, SAT, and VAT/SAT ratio, and mmol/L increase in total cholesterol, HDL, LDL, triglycerides, and ratio TG/HDL. Model 1 = adjusted for sex and age; Model 2 = Model 1 + BSA, smoking, alcohol use, diabetes, hypertension; Model 3 = Model 2 + glucose, insulin, GGT, AST, ALP, creatinine; Model 4 = Model 3 + lipid lowering medication and physical activity. CI = 95% confidence interval; Abbreviation: BSA= body surface area; HDL = high-density lipoprotein; LDL = low-density lipoprotein; AST = aspartate transaminase; ALP = alkaline phosphatase; GGT = γ-glutamyl transpeptidase; TAT = total adipose tissue; TG = triglycerides; VAT = visceral adipose tissue; SAT = subcutaneous adipose tissue.

## Data Availability

The informed consent given by KORA study participants does not cover data posting in public databases. However, data are available upon request by means of a project agreement. Requests should be sent to kora.passt@helmholtz-muenchen.de and are subject to approval by the KORA Board.

## Supplementary Materials

The following supporting information can be downloaded at: https://www.mdpi.com/article/10.3390/nu14040797/s1, Table S1: Lipid profile of study population according to tertiles of BMI; Tables S2 and S3: Association between TAT, VAT, SAT, and VAT/SAT ratio with total cholesterol, HDL, LDL, triglycerides, and TG/HDL ratio according to sex; Tables S4–S6: Association between TAT, VAT, SAT, and VAT/SAT ratio with total cholesterol, HDL, LDL, triglycerides, and TG/HDL ratio according to BMI categories.
