# Supplementary material for: Association between Adipose Tissue Depots and Dyslipidemia: The KORA-MRI Population-Based Study
Source: Nutrients. 2022 Feb 14;14(4):797. doi: 10.3390/nu14040797 (PMC8879798; doi:10.3390/nu14040797)
Supplement: Supplementary file 1 [file nutrients-14-00797-s001.zip › nutrients-1580910-supplementary.pdf]

SUPPLEMENTARY MATERIAL

Table S1 Lipid profile of study population according to tertiles of BMI

**BMI**	**Low tertile < 25.6**<br>**N=129**	**Medium tertile 25.6 – 29.2**<br>**N=127**	**High tertile > 29.2**<br>**N=128**	**P**
Total cholesterol	5.62 (±0.99)	5.68 (±0.93)	5.58 (±0.88)	0.904
HDL	1.79 (±0.48)	1.60 (±0.44)	1.41 (±0.37)	<0.001
LDL	3.53 (±0.92)	3.65 (±0.81)	3.63 (±0.79)	0.215
Triglycerides	0.93 (0.75;1.28)	1.19 (0.94;1.61)	1.58 (1.17;2.27)	<0.001
Ratio TG/HDL	0.55 (0.39;0.89)	0.78 (0.52;1.19)	1.18 (0.69;1.89)	<0.001
TAT	7.35 (5.97;9.7)	11.31 (9.75;13.6)	17.45 (15.51;20.02)	<0.001
VAT	1.97 (1.28;3.44)	3.99 (2.99;5.48)	6.30 (4.6;8.34)	<0.001
SAT	5.14 (4.31;6.02)	6.96 (5.84;8.72)	10.93 (8.78;13.74)	<0.001
Ratio VAT/SAT	0.40 (0.23;0.68)	0.61 (0.37;0.84)	0.57 (0.35;0.87)	<0.001

Values are represented as mean with standard deviation (SD) or median with interquartile range (IQR); P- depicts for trend. Abbreviation: HDL =high-density lipoprotein; LDL = low-density lipoprotein; TG = triglycerides

Table S2 Association between TAT, VAT, SAT, and VAT/SAT ratio with total cholesterol, HDL, LDL, triglycerides, and TG/HDL ratio according to sex

**Female**	**Total cholesterol**							
Per SD log transformed	**Model 1**	**p-value**	**Model 2**	**p-value**	**Model 3**	**p-value**	**Model 4**	**p-value**
TAT	0.02 (-0.10; 0.15)	0.697	0.37 (0.18; 0.56)	<0.001	0.43 (0.19; 0.66)	<0.001	0.44 (0.22; 0.67)	<0.001
VAT	0.10 (-0.04; 0.24)	0.159	0.39 (0.20; 0.57)	<0.001	0.42 (0.20; 0.64)	<0.001	0.44 (0.23; 0.65)	<0.001
SAT	0.01 (-0.11; 0.14)	0.845	0.34 (0.14; 0.54)	0.001	0.38 (0.15; 0.61)	0.002	0.37 (0.14; 0.60)	0.002
Ratio VAT/SAT	0.03 (-0.14; 0.21)	0.697	0.53 (0.25; 0.8)	<0.001	0.61 (0.28; 0.95)	<0.001	0.64 (0.31; 0.96)	<0.001
	**HDL**							
TAT	-0.18 (-0.24; -0.12)	<0.001	-0.19 (-0.29; -0.10)	<0.001	-0.15 (-0.27; -0.03)	0.013	-0.15 (-0.27; -0.04)	0.012
VAT	-0.23 (-0.30; -0.16)	<0.001	-0.21 (-0.31; -0.12)	<0.001	-0.19 (-0.30; -0.08)	0.001	-0.19 (-0.30; -0.08)	0.001
SAT	-0.17 (-0.23; -0.10)	<0.001	-0.16 (-0.25; -0.06)	0.002	-0.09 (-0.21; 0.02)	0.120	-0.10 (-0.21; 0.02)	0.113
Ratio VAT/SAT	-0.25 (-0.34; -0.17)	<0.001	-0.28 (-0.41; -0.14)	<0.001	-0.21 (-0.38; -0.05)	0.013	-0.22 (-0.39; -0.05)	0.012
	**LDL**							
TAT	0.11 (0.00; 0.22)	0.060	0.39 (0.21; 0.57)	<0.001	0.44 (0.22; 0.67)	<0.001	0.46 (0.25; 0.67)	<0.001
VAT	0.20 (0.07; 0.33)	0.003	0.43 (0.26; 0.60)	<0.001	0.47 (0.27; 0.68)	<0.001	0.49 (0.30; 0.69)	<0.001
SAT	0.10 (-0.02; 0.21)	0.110	0.34 (0.16; 0.53)	<0.001	0.36 (0.14; 0.58)	0.001	0.35 (0.14; 0.57)	0.002
Ratio VAT/SAT	0.16 (-0.01; 0.32)	0.060	0.56 (0.31; 0.82)	<0.001	0.64 (0.32; 0.95)	<0.001	0.66 (0.35; 0.96)	<0.001
	**Triglycerides**							
TAT	0.14 (0.09; 0.20)	<0.001	0.24 (0.16; 0.33)	<0.001	0.20 (0.09; 0.30)	<0.001	0.20 (0.09; 0.31)	<0.001
VAT	0.21 (0.15; 0.27)	<0.001	0.28 (0.20; 0.36)	<0.001	0.25 (0.15; 0.35)	<0.001	0.25 (0.15; 0.35)	<0.001
SAT	0.13 (0.07; 0.18)	<0.001	0.20 (0.11; 0.30)	<0.001	0.14 (0.03; 0.25)	0.011	0.14 (0.03; 0.25)	0.011
Ratio VAT/SAT	0.21 (0.13; 0.28)	<0.001	0.35 (0.22; 0.47)	<0.001	0.28 (0.12; 0.43)	<0.001	0.29 (0.13; 0.44)	<0.001
	**Ratio TG/HDL**							
TAT	0.24 (0.16; 0.32)	<0.001	0.35 (0.22; 0.47)	<0.001	0.27 (0.12; 0.42)	0.001	0.28 (0.12; 0.43)	0.001
VAT	0.33 (0.25; 0.42)	<0.001	0.39 (0.28; 0.51)	<0.001	0.34 (0.21; 0.48)	<0.001	0.35 (0.21; 0.49)	<0.001
SAT	0.22 (0.14; 0.30)	<0.001	0.28 (0.15; 0.42)	<0.001	0.19 (0.03; 0.34)	0.016	0.19 (0.04; 0.35)	0.016
Ratio VAT/SAT	0.34 (0.23; 0.45)	<0.001	0.49 (0.32; 0.67)	<0.001	0.39 (0.17; 0.6)	0.001	0.40 (0.17; 0.62)	0.001

The beta estimate from linear regression model given with a 95% confidence interval represents the estimate size per SD of TAT, VAT and SAT and mmol/L increase in total cholesterol, HDL, LDL and triglycerides. Model 1 = adjusted for sex and age; Model 2 = Model 1 + BSA, smoking, alcohol use, diabetes, hypertension; Model 3 = Model 2 + glucose, insulin, GGT, AST, ALP, creatinine; Model 4 = Model 3 + lipid lowering medication and physical activity.
CI = 95% confidence interval; Abbreviation: BSA= body surface area; HDL = high-density lipoprotein; LDL = low-density lipoprotein; AST = aspartate transaminase; ALP = alkaline phosphatase; GGT = gamma glutamyl transpeptidase; TAT = total adipose tissue; TG = triglycerides; VAT = visceral adipose tissue; SAT = subcutaneous adipose tissue.

Table S3 Association between TAT, VAT, SAT, and VAT/SAT ratio with total cholesterol, HDL, LDL, triglycerides, and TG/HDL ratio according to sex

**Male**	**Total cholesterol**							
Per SD log transformed	**Model 1**	**p-value**	**Model 2**	**p-value**	**Model 3**	**p-value**	**Model 4**	**p-value**
TAT	0.05 (-0.10; 0.19)	0.522	0.32 (0.11; 0.52)	0.003	0.25 (0.02; 0.49)	0.037	0.27 (0.04; 0.51)	0.020
VAT	0.15 (-0.05; 0.35)	0.141	0.38 (0.14; 0.62)	0.002	0.29 (0.02; 0.57)	0.035	0.30 (0.04; 0.56)	0.026
SAT	0.00 (-0.14; 0.15)	0.957	0.25 (0.05; 0.46)	0.014	0.20 (-0.02; 0.43)	0.079	0.22 (0.00; 0.44)	0.048
Ratio VAT/SAT	0.07 (-0.14; 0.28)	0.522	0.45 (0.16; 0.74)	0.003	0.36 (0.02; 0.71)	0.037	0.39 (0.06; 0.72)	0.020
	**HDL**							
TAT	-0.15 (-0.21; -0.10)	<0.001	-0.11 (-0.19; -0.04)	0.004	-0.03 (-0.11; 0.06)	0.493	-0.03 (-0.11; 0.06)	0.524
VAT	-0.21 (-0.29; -0.14)	<0.001	-0.17 (-0.26; -0.09)	<0.001	-0.10 (-0.19; 0.00)	0.044	-0.10 (-0.20; 0.00)	0.046
SAT	-0.12 (-0.18; -0.07)	<0.001	-0.05 (-0.13; 0.02)	0.156	0.02 (-0.06; 0.10)	0.548	0.03 (-0.05; 0.11)	0.516
Ratio VAT/SAT	-0.22 (-0.29; -0.14)	<0.001	-0.16 (-0.26; -0.05)	0.004	-0.04 (-0.16; 0.08)	0.493	-0.04 (-0.16; 0.08)	0.524
	**LDL**							
TAT	0.04 (-0.09; 0.17)	0.523	0.22 (0.04; 0.41)	0.017	0.18 (-0.04; 0.40)	0.105	0.20 (-0.01; 0.41)	0.064
VAT	0.10 (-0.08; 0.27)	0.267	0.28 (0.06; 0.49)	0.013	0.21 (-0.04; 0.46)	0.097	0.21 (-0.02; 0.45)	0.077
SAT	0.02 (-0.10; 0.15)	0.728	0.18 (0.00; 0.36)	0.052	0.14 (-0.06; 0.35)	0.169	0.16 (-0.04; 0.36)	0.111
Ratio VAT/SAT	0.06 (-0.12; 0.24)	0.523	0.32 (0.06; 0.58)	0.017	0.26 (-0.05; 0.57)	0.105	0.28 (-0.02; 0.58)	0.064
	**Triglycerides**							
TAT	0.31 (0.23; 0.38)	<0.001	0.34 (0.24; 0.44)	<0.001	0.21 (0.10; 0.32)	<0.001	0.21 (0.10; 0.32)	<0.001
VAT	0.48 (0.38; 0.57)	<0.001	0.47 (0.35; 0.58)	<0.001	0.34 (0.21; 0.46)	<0.001	0.34 (0.21; 0.46)	<0.001
SAT	0.22 (0.14; 0.30)	<0.001	0.21 (0.11; 0.32)	<0.001	0.07 (-0.04; 0.18)	0.186	0.07 (-0.04; 0.18)	0.204
Ratio VAT/SAT	0.44 (0.33; 0.54)	<0.001	0.49 (0.35; 0.64)	<0.001	0.30 (0.14; 0.46)	<0.001	0.30 (0.14; 0.46)	<0.001
	**Ratio TG/HDL**							
TAT	0.42 (0.32; 0.52)	<0.001	0.43 (0.29; 0.56)	<0.001	0.23 (0.08; 0.38)	0.003	0.23 (0.07; 0.38)	0.004
VAT	0.64 (0.51; 0.76)	<0.001	0.59 (0.43; 0.75)	<0.001	0.40 (0.24; 0.57)	<0.001	0.40 (0.23; 0.57)	<0.001
SAT	0.31 (0.21; 0.41)	<0.001	0.25 (0.11; 0.40)	0.001	0.06 (-0.09; 0.20)	0.455	0.05 (-0.10; 0.20)	0.493
Ratio VAT/SAT	0.60 (0.46; 0.74)	<0.001	0.61 (0.41; 0.81)	<0.001	0.33 (0.11; 0.54)	0.003	0.32 (0.11; 0.54)	0.004

The beta estimate from linear regression model given with a 95% confidence interval represents the estimate size per SD of TAT, VAT and SAT and mmol/L increase in total cholesterol, HDL, LDL and triglycerides. Model 1 = adjusted for sex and age; Model 2 = Model 1 + BSA, smoking, alcohol use, diabetes, hypertension; Model 3 = Model 2 + glucose, insulin, GGT, AST, ALP, creatinine; Model 4 = Model 3 + lipid lowering medication and physical activity.
CI = 95% confidence interval; Abbreviation: BSA= body surface area; HDL = high-density lipoprotein; LDL = low-density lipoprotein; AST = aspartate transaminase; ALP = alkaline phosphatase; GGT = gamma glutamyl transpeptidase; TAT = total adipose tissue; TG = triglycerides; VAT = visceral adipose tissue; SAT = subcutaneous adipose tissue.

Table S4 Association between TAT, VAT, SAT, and VAT/SAT ratio with total cholesterol, HDL, LDL, triglycerides, and TG/HDL ratio according to BMI categories

**BMI <25.6 (N=129)**	**Total cholesterol**							
Per SD log transformed	**Model 1**	**p-value**	**Model 2**	**p-value**	**Model 3**	**p-value**	**Model 4**	**p-value**
TAT	0.35 (0.11; 0.59)	0.005	0.52 (0.25; 0.79)	<0.001	0.27 (-0.03; 0.58)	0.078	0.26 (-0.03; 0.56)	0.083
VAT	0.47 (0.21; 0.73)	<0.001	0.55 (0.27; 0.83)	<0.001	0.35 (0.05; 0.65)	0.021	0.32 (0.03; 0.61)	0.032
SAT	0.29 (0.05; 0.53)	0.019	0.50 (0.23; 0.78)	<0.001	0.27 (-0.03; 0.57)	0.075	0.25 (-0.04; 0.54)	0.094
Ratio VAT/SAT	0.5 (0.15; 0.84)	0.005	0.75 (0.36; 1.13)	<0.001	0.39 (-0.04; 0.83)	0.078	0.37 (-0.05; 0.8)	0.083
	**HDL**							
TAT	-0.18 (-0.29; -0.07)	0.002	-0.22 (-0.33; -0.11)	<0.001	-0.18 (-0.3; -0.05)	0.006	-0.18 (-0.31; -0.06)	0.005
VAT	-0.27 (-0.39; -0.16)	<0.001	-0.27 (-0.38; -0.17)	<0.001	-0.25 (-0.37; -0.13)	<0.001	-0.25 (-0.37; -0.13)	<0.001
SAT	-0.11 (-0.23; 0)	0.049	-0.16 (-0.27; -0.05)	0.006	-0.1 (-0.23; 0.02)	0.108	-0.11 (-0.23; 0.02)	0.097
Ratio VAT/SAT	-0.25 (-0.41; -0.1)	0.002	-0.31 (-0.47; -0.16)	<0.001	-0.25 (-0.43; -0.07)	0.006	-0.26 (-0.44; -0.08)	0.005
	**LDL**							
TAT	0.36 (0.14; 0.58)	0.002	0.53 (0.28; 0.78)	<0.001	0.34 (0.05; 0.62)	0.023	0.33 (0.05; 0.61)	0.023
VAT	0.51 (0.28; 0.75)	<0.001	0.59 (0.33; 0.85)	<0.001	0.45 (0.17; 0.73)	0.002	0.42 (0.14; 0.69)	0.003
SAT	0.3 (0.07; 0.52)	0.011	0.49 (0.23; 0.75)	<0.001	0.29 (0.01; 0.57)	0.045	0.27 (-0.01; 0.54)	0.056
Ratio VAT/SAT	0.51 (0.2; 0.83)	0.002	0.76 (0.4; 1.12)	<0.001	0.48 (0.07; 0.89)	0.023	0.47 (0.07; 0.86)	0.023
	**Triglycerides**							
TAT	0.31 (0.21; 0.42)	<0.001	0.38 (0.27; 0.5)	<0.001	0.26 (0.13; 0.38)	<0.001	0.26 (0.14; 0.39)	<0.001
VAT	0.43 (0.31; 0.54)	<0.001	0.43 (0.32; 0.55)	<0.001	0.32 (0.21; 0.44)	<0.001	0.33 (0.21; 0.44)	<0.001
SAT	0.21 (0.1; 0.33)	<0.001	0.3 (0.17; 0.43)	<0.001	0.16 (0.04; 0.29)	0.009	0.17 (0.05; 0.3)	0.006
Ratio VAT/SAT	0.45 (0.29; 0.61)	<0.001	0.55 (0.38; 0.72)	<0.001	0.36 (0.19; 0.54)	<0.001	0.38 (0.2; 0.55)	<0.001
	**Ratio TG/HDL**							
TAT	0.42 (0.27; 0.56)	<0.001	0.5 (0.35; 0.65)	<0.001	0.34 (0.18; 0.51)	<0.001	0.36 (0.19; 0.52)	<0.001
VAT	0.58 (0.43; 0.73)	<0.001	0.59 (0.44; 0.73)	<0.001	0.45 (0.30; 0.6)	<0.001	0.46 (0.31; 0.61)	<0.001
SAT	0.28 (0.12; 0.43)	0.001	0.38 (0.22; 0.55)	<0.001	0.21 (0.05; 0.38)	0.013	0.22 (0.06; 0.39)	0.009
Ratio VAT/SAT	0.59 (0.39; 0.8)	<0.001	0.72 (0.5; 0.93)	<0.001	0.49 (0.26; 0.72)	<0.001	0.51 (0.28; 0.74)	<0.001

The beta estimate from linear regression model given with a 95% confidence interval represents the estimate size per SD of TAT, VAT and SAT and mmol/L increase in total cholesterol, HDL, LDL and triglycerides. Model 1 = adjusted for sex and age; Model 2 = Model 1 + BSA, smoking, alcohol use, diabetes, hypertension; Model 3 = Model 2 + glucose, insulin, GGT, AST, ALP, creatinine; Model 4 = Model 3 + lipid lowering medication and physical activity.

CI = 95% confidence interval; Abbreviation: BSA= body surface area; HDL = high-density lipoprotein; LDL = low-density lipoprotein; AST = aspartate transaminase; ALP = alkaline phosphatase; GGT = gamma glutamyl transpeptidase; TAT = total adipose tissue; TG = triglycerides; VAT = visceral adipose tissue; SAT = subcutaneous adipose tissue.

Table S5 Association between TAT, VAT, SAT, and VAT/SAT ratio with total cholesterol, HDL, LDL, triglycerides, and TG/HDL ratio according to BMI categories

**BMI 25.6 – 29.2 (N=127)**	**Total cholesterol**							
Per SD log transformed	**Model 1**	**p-value**	**Model 2**	**p-value**	**Model 3**	**p-value**	**Model 4**	**p-value**
TAT	0.36 (0.05; 0.68)	0.025	0.58 (0.21; 0.94)	0.002	0.55 (0.12; 0.98)	0.014	0.53 (0.11; 0.96)	0.015
VAT	0.39 (0.12; 0.66)	0.005	0.49 (0.2; 0.78)	0.001	0.42 (0.08; 0.76)	0.016	0.44 (0.11; 0.77)	0.010
SAT	0.33 (-0.02; 0.68)	0.066	0.52 (0.11; 0.93)	0.012	0.55 (0.12; 0.99)	0.014	0.52 (0.09; 0.95)	0.018
Ratio VAT/SAT	0.52 (0.07; 0.96)	0.025	0.82 (0.3; 1.34)	0.002	0.78 (0.16; 1.4)	0.014	0.76 (0.15; 1.37)	0.015
	**HDL**							
TAT	-0.13 (-0.26; 0.01)	0.068	-0.07 (-0.23; 0.09)	0.366	-0.06 (-0.24; 0.12)	0.504	-0.07 (-0.25; 0.12)	0.473
VAT	-0.16 (-0.27; -0.04)	0.009	-0.13 (-0.26; -0.01)	0.039	-0.13 (-0.26; 0.01)	0.072	-0.14 (-0.28; 0.00)	0.054
SAT	-0.05 (-0.2; 0.11)	0.557	0.05 (-0.12; 0.23)	0.541	0.08 (-0.1; 0.26)	0.382	0.08 (-0.10; 0.26)	0.382
Ratio VAT/SAT	-0.18 (-0.38; 0.01)	0.068	-0.10 (-0.33; 0.12)	0.366	-0.09 (-0.34; 0.17)	0.504	-0.09 (-0.35; 0.16)	0.473
	**LDL**							
TAT	0.33 (0.06; 0.6)	0.019	0.45 (0.14; 0.76)	0.005	0.54 (0.17; 0.92)	0.005	0.52 (0.16; 0.89)	0.005
VAT	0.33 (0.1; 0.57)	0.006	0.41 (0.16; 0.66)	0.001	0.43 (0.14; 0.72)	0.004	0.45 (0.17; 0.74)	0.002
SAT	0.34 (0.04; 0.65)	0.028	0.38 (0.03; 0.73)	0.032	0.46 (0.08; 0.85)	0.018	0.43 (0.06; 0.80)	0.024
Ratio VAT/SAT	0.47 (0.08; 0.86)	0.019	0.64 (0.20; 1.09)	0.005	0.78 (0.24; 1.32)	0.005	0.75 (0.23; 1.27)	0.005
	**Triglycerides**							
TAT	0.35 (0.19; 0.51)	<0.001	0.34 (0.15; 0.52)	<0.001	0.19 (-0.02; 0.39)	0.078	0.19 (-0.02; 0.40)	0.072
VAT	0.38 (0.25; 0.52)	<0.001	0.34 (0.2; 0.49)	<0.001	0.22 (0.07; 0.38)	0.006	0.23 (0.07; 0.39)	0.006
SAT	0.19 (0.01; 0.38)	0.044	0.18 (-0.03; 0.39)	0.099	0.07 (-0.14; 0.28)	0.487	0.08 (-0.13; 0.29)	0.458
Ratio VAT/SAT	0.5 (0.27; 0.73)	<0.001	0.48 (0.22; 0.75)	<0.001	0.26 (-0.03; 0.56)	0.078	0.27 (-0.02; 0.57)	0.072
	**Ratio TG/HDL**							
TAT	0.45 (0.23; 0.66)	<0.001	0.41 (0.15; 0.66)	0.002	0.25 (-0.03; 0.53)	0.085	0.26 (-0.03; 0.54)	0.077
VAT	0.49 (0.31; 0.67)	<0.001	0.44 (0.24; 0.64)	<0.001	0.31 (0.1; 0.53)	0.005	0.33 (0.11; 0.55)	0.004
SAT	0.23 (-0.02; 0.48)	0.073	0.16 (-0.13; 0.45)	0.276	0.04 (-0.25; 0.32)	0.796	0.04 (-0.25; 0.33)	0.771
Ratio VAT/SAT	0.64 (0.33; 0.95)	<0.001	0.58 (0.22; 0.94)	0.002	0.35 (-0.05; 0.76)	0.085	0.37 (-0.04; 0.77)	0.077

The beta estimate from linear regression model given with a 95% confidence interval represents the estimate size per SD of TAT, VAT and SAT and mmol/L increase in total cholesterol, HDL, LDL and triglycerides. Model 1 = adjusted for sex and age; Model 2 = Model 1 + BSA, smoking, alcohol use, diabetes, hypertension; Model 3 = Model 2 + glucose, insulin, GGT, AST, ALP, creatinine; Model 4 = Model 3 + lipid lowering medication and physical activity.
CI = 95% confidence interval; Abbreviation: BSA= body surface area; HDL = high-density lipoprotein; LDL = low-density lipoprotein; AST = aspartate transaminase; ALP = alkaline phosphatase; GGT = gamma glutamyl transpeptidase; TAT = total adipose tissue; TG = triglycerides; VAT = visceral adipose tissue; SAT = subcutaneous adipose tissue.

Table S6 Association between TAT, VAT, SAT, and VAT/SAT ratio with total cholesterol, HDL, LDL, triglycerides, and TG/HDL ratio according to BMI categories

**BMI > 29.2 (N=128)**	**Total cholesterol**							
Per SD log transformed	**Model 1**	**p-value**	**Model 2**	**p-value**	**Model 3**	**p-value**	**Model 4**	**p-value**
TAT	-0.24 (-0.53; 0.05)	0.098	0.06 (-0.29; 0.41)	0.729	0.06 (-0.33; 0.44)	0.773	0.02 (-0.35; 0.39)	0.916
VAT	-0.10 (-0.44; 0.24)	0.568	0.06 (-0.30; 0.42)	0.733	0.04 (-0.34; 0.43)	0.820	0.06 (-0.32; 0.43)	0.756
SAT	-0.21 (-0.45; 0.04)	0.095	0.07 (-0.23; 0.36)	0.659	0.07 (-0.25; 0.39)	0.685	0.00 (-0.31; 0.32)	0.977
Ratio VAT/SAT	-0.35 (-0.76; 0.06)	0.098	0.09 (-0.41; 0.58)	0.729	0.08 (-0.46; 0.62)	0.773	0.03 (-0.50; 0.56)	0.916
	**HDL**							
TAT	-0.08 (-0.19; 0.03)	0.133	-0.06 (-0.20; 0.07)	0.358	0.00 (-0.13; 0.14)	0.955	0.01 (-0.13; 0.15)	0.883
VAT	-0.05 (-0.17; 0.08)	0.461	-0.03 (-0.17; 0.11)	0.652	0.04 (-0.10; 0.18)	0.598	0.05 (-0.09; 0.19)	0.480
SAT	-0.06 (-0.15; 0.03)	0.187	-0.03 (-0.14; 0.08)	0.612	0.01 (-0.10; 0.13)	0.815	0.01 (-0.11; 0.13)	0.858
Ratio VAT/SAT	-0.12 (-0.27; 0.04)	0.133	-0.09 (-0.28; 0.10)	0.358	0.01 (-0.19; 0.20)	0.955	0.01 (-0.19; 0.22)	0.883
	**LDL**							
TAT	-0.28 (-0.54; -0.02)	0.033	-0.01 (-0.32; 0.30)	0.951	-0.02 (-0.36; 0.33)	0.920	-0.05 (-0.39; 0.29)	0.772
VAT	-0.24 (-0.54; 0.06)	0.120	-0.06 (-0.38; 0.27)	0.724	-0.08 (-0.43; 0.27)	0.663	-0.07 (-0.41; 0.28)	0.691
SAT	-0.20 (-0.42; 0.02)	0.080	0.04 (-0.23; 0.30)	0.791	0.03 (-0.26; 0.32)	0.819	-0.02 (-0.30; 0.27)	0.906
Ratio VAT/SAT	-0.40 (-0.77; -0.03)	0.033	-0.01 (-0.46; 0.44)	0.951	-0.03 (-0.52; 0.47)	0.920	-0.07 (-0.56; 0.42)	0.772
	**Triglycerides**							
TAT	0.15 (0.00; 0.30)	0.053	0.16 (-0.02; 0.34)	0.079	0.07 (-0.11; 0.25)	0.449	0.06 (-0.13; 0.25)	0.547
VAT	0.29 (0.13; 0.46)	0.001	0.26 (0.08; 0.44)	0.006	0.19 (0.00; 0.37)	0.048	0.17 (-0.01; 0.36)	0.069
SAT	0.04 (-0.09; 0.16)	0.587	0.04 (-0.12; 0.19)	0.638	-0.04 (-0.19; 0.12)	0.620	-0.05 (-0.20; 0.11)	0.565
Ratio VAT/SAT	0.21 (0.00; 0.42)	0.053	0.23 (-0.03; 0.49)	0.079	0.1 (-0.16; 0.36)	0.449	0.08 (-0.19; 0.35)	0.547
	**Ratio TG/HDL**							
TAT	0.21 (0.01; 0.42)	0.042	0.21 (-0.05; 0.46)	0.11	0.06 (-0.19; 0.32)	0.631	0.04 (-0.22; 0.30)	0.741
VAT	0.33 (0.10; 0.57)	0.006	0.28 (0.02; 0.54)	0.033	0.16 (-0.1; 0.42)	0.228	0.14 (-0.12; 0.40)	0.302
SAT	0.09 (-0.09; 0.26)	0.341	0.06 (-0.16; 0.27)	0.605	-0.05 (-0.27; 0.16)	0.623	-0.06 (-0.28; 0.16)	0.595
Ratio VAT/SAT	0.31 (0.01; 0.60)	0.042	0.29 (-0.07; 0.66)	0.110	0.09 (-0.28; 0.45)	0.631	0.06 (-0.31; 0.43)	0.741

The beta estimate from linear regression model given with a 95% confidence interval represents the estimate size per SD of TAT, VAT and SAT and mmol/L increase in total cholesterol, HDL, LDL and triglycerides. Model 1 = adjusted for sex and age; Model 2 = Model 1 + BSA, smoking, alcohol use, diabetes, hypertension; Model 3 = Model 2 + glucose, insulin, GGT, AST, ALP, creatinine; Model 4 = Model 3 + lipid lowering medication and physical activity.
CI = 95% confidence interval; Abbreviation: BSA= body surface area; HDL = high-density lipoprotein; LDL = low-density lipoprotein; AST = aspartate transaminase; ALP = alkaline phosphatase; GGT = gamma glutamyl transpeptidase; TAT = total adipose tissue; TG = triglycerides; VAT = visceral adipose tissue; SAT = subcutaneous adipose tissue.
